# A Single Transcriptome of a Green Toad (*Bufo viridis*) Yields Candidate Genes for Sex Determination and -Differentiation and Non-Anonymous Population Genetic Markers

**DOI:** 10.1371/journal.pone.0156419

**Published:** 2016-05-27

**Authors:** Jörn F. Gerchen, Samuel J. Reichert, Johannes T. Röhr, Christoph Dieterich, Werner Kloas, Matthias Stöck

**Affiliations:** 1 Department of Ecophysiology and Aquaculture, Leibniz-Institute of Freshwater Ecology and Inland Fisheries (IGB), Berlin, Germany; 2 Leibniz Institute for Research on Evolution and Biodiversity, Berlin, Germany; 3 Department of Mathematics and Computer Science, Freie Universität Berlin, Berlin, Germany; 4 Max Planck Institute for Biology of Ageing, Cologne, Germany; State Natural History Museum, GERMANY

## Abstract

Large genome size, including immense repetitive and non-coding fractions, still present challenges for capacity, bioinformatics and thus affordability of whole genome sequencing in most amphibians. Here, we test the performance of a single transcriptome to understand whether it can provide a cost-efficient resource for species with large unknown genomes. Using RNA from six different tissues from a single Palearctic green toad (*Bufo viridis*) specimen and Hiseq2000, we obtained 22,5 Mio reads and publish >100,000 unigene sequences. To evaluate efficacy and quality, we first use this data to identify green toad specific candidate genes, known from other vertebrates for their role in sex determination and differentiation. Of a list of 37 genes, the transcriptome yielded 32 (87%), many of which providing the first such data for this non-model anuran species. However, for many of these genes, only fragments could be retrieved. In order to allow also applications to population genetics, we further used the transcriptome for the targeted development of 21 non-anonymous microsatellites and tested them in genetic families and backcrosses. Eleven markers were specifically developed to be located on the *B*. *viridis* sex chromosomes; for eight markers we can indeed demonstrate sex-specific transmission in genetic families. Depending on phylogenetic distance, several markers, which are sex-linked in green toads, show high cross-amplification success across the anuran phylogeny, involving nine systematic anuran families. Our data support the view that single transcriptome sequencing (based on multiple tissues) provides a reliable genomic resource and cost-efficient method for non-model amphibian species with large genome size and, despite limitations, should be considered as long as genome sequencing remains unaffordable for most species.

## Introduction

### Rare availability of amphibian genomes

The availability of whole genome sequencing has greatly facilitated evolutionary studies in non-model organisms [1]. Besides comparative research involving entire genomes (e.g. [2]), applications include candidate gene approaches and the development of specific marker sets for population genetics and speciation research. However, for some groups of non-model organisms, like most amphibians with very high DNA content [3], whole genome sequencing still is bioinformatically challenging and costly, and very few complete genomes are available. Due to their large proportion of repetitive sequences, assembly and proper annotation of such genomes remain difficult. Currently only two assembled and annotated amphibian genomes have become available: that of the model anuran, the tropical clawed frog, *Xenopus tropicalis* (Pipidae) [4], and recently that of the Tibetan frog, *Nanorana parkeri* (Dicroglossidae) [5].

### Transcriptome sequencing as genomic resource for large-genome non-model species

Genetic and genomic applications often involve candidate gene approaches, in which the role of genes, known from related or model species, is examined in association studies for non-model species. In taxa with huge, unknown genomes, where whole genome sequencing still remains unaffordable, a useful resource may be transcriptomics (e.g. [6]). As transcriptomes only include exons and UTRs, sequencing efforts can be restricted to a small portion of the genome, and thus to the exclusion of hard-to-assemble non-coding intronic and intergenic repetitive regions. Amphibian transcriptomic information is expected to be well-annotatable. Given that many restrictions for whole genome approaches include most amphibians, in the present paper, we aimed at testing the usefulness of a single anuran transcriptome for obtaining a list of candidate genes on sexual development and sex determination.

As another application, we aimed at the targeted development of non-anonymous transcriptomic microsatellites [7, 8], namely sex-specific and sex-linked markers. They appear less susceptible to phylogenetic noise than SNPs [9] and are applicable to population genetics and to study inheritance patterns [10, 11].

As our target species, we have chosen a diploid bufonid toad, the European green toad *Bufo viridis*. Importantly, no genome or transcriptome of the species or of related taxa from this systematic anuran family have so far been characterized. Palearctic green toads (*B*. *viridis* subgroup) form a complex of at least 12 mitochondrial lineages that inhabit large parts of Europe, North Africa and Asia with several diploid clades as well as tri- and tetraploid lineages [12]. Multiple natural hybrid zones of lineages with different divergence time can be found in this group [13, 14], making it a highly attractive amphibian radiation to study speciation and sex chromosome evolution under diploid [15] and polyploid hybridization [16]. In this paper, we present 11 markers located on the sex chromosome (with sex-linkage proven for 8 markers) and 10 autosomal markers for the *B*. *viridis* subgroup, most of which are transcriptome based and polymorphic.

## Material and Methods

### Animal sampling / ethics statement

Our study was approved by the relevant Institutional Animal Care and Use Committee (IACUC), namely the Service de la Consommation et des Affaires Vétérinaires du Canton de Vaud (Epalinges, Switzerland); toads and/or clutches were sampled according to permit 115790/229 in accordance with the Swiss import permit of the Bundesamt für Veterinärwesen, BVET Nr. 812/11, Bern, Switzerland, and authorization No. 1798, Service de la consommation et des affaires vétérinaires, Canton de Vaud, Epalinges, Switzerland.

### Sequencing of a single transcriptome

RNA was extracted from six different tissues (liver, eye, brain, testes, heart, muscle) of a single male *Bufo viridis*, caught south of Astros, Greece (37.38299 N, 22.7455 E) in March 2011. RNA was pooled and adjusted to equal concentrations. Complementary DNA (cDNA) was synthesized and sequenced by BGI (BGI-Hongkong Co., Ltd.), using the Hiseq2000 sequencing system (Illumina, San Diego, USA). Reads were assembled using SOAPdenovo [17]: contigs (longest assembled sequences without Ns) were linked into scaffolds by mapping reads back to contigs and combining paired-end information. Scaffold length was estimated according to the average segment length of each pair of reads, and unknown bases were filled with Ns. Gaps in scaffolds were filled using paired-end reads, resulting in so-called unigenes. Raw reads were transmitted to the NCBI Sequence Read Archive (SRA, http://www.ncbi.nlm.nih.gov/Traces/sra/) under the accession Number SRS1034407, assembled sequences were transmitted to NCBI Transcriptome Shotgun Assembly Sequence Database (TSA, http://www.ncbi.nlm.nih.gov/genbank/tsa), BioSample accession for the project is SAMN03993917.

### Functional annotation and classification

To predict protein functional information from the annotation of blast hits, unigene sequences were searched against four protein databases (Nr, Swissprot, KEGG, COG) using Blastx [18] with an *E-value* threshold of E-05. Blast2GO [19] as applied for GO annotation on the basis of the results of the Nr annotation. GO functional classification of all unigenes was performed using WEGO [20].

### Genes involved in sex determination or sex differentiation

To evaluate the usefulness of a single, though multiple tissue-based, amphibian transcriptome for candidate gene approaches, we aimed at retrieving genes involved in male or female sex determination and sexual differentiation (list from M. Schartl, pers. comm., modified for anurans). Template sequences from the anuran model species *Xenopus tropicalis* or *X*. *laevis* were retrieved from Xenbase [21]. Sequences were blasted against a local database of all *B*. *viridis* unigenes using tblastn [18]. Using NCBI blastn, homology of unigenes from the resulting blast hits was confirmed. Only best blast hits with an *E-value* lower than E-05 were considered homologous. Blast hits were not considered homologous when there were other blast hits for different genes with E-values less then 10000 times larger.

### Development of transcriptome-based microsatellite markers

Despite 150 million years (My) of divergence between bufonid, hylid and ranid anurans, chromosome number and interphase banding patterns suggest a high degree of structural conservation of their genomes [22]. Even after 200 My of separation, homology between large parts of *X*. *tropicalis* chromosome 1 (in which it is not the sex chromosome) and the sex chromosome of all examined diploid species of the *B*. *viridis* subgroup has been shown [15, 23]. Lacking more closely related relatives, the genome of *X*. *tropicalis* (and *X*. *laevis*) served as reference for *B*. *viridis*. Transcriptome data were first searched for microsatellite-like repetitive sequences (SSRs) using Misa (MIcroSAtellite identification tool, URL: http://pgrc.ipk-gatersleben.de/misa/). Repeats were filtered to exclude short repeats and duplicate entries. Sequences of repeat-containing unigenes were searched against the transcriptome of *X*. *tropicalis* [4] using the local alignment software Blast+ 2.2.25 [18] with the options "–evalue = 0.01 and –task = blastn" to identify homologous transcripts in *X*. *tropicalis*. The resulting best blast hits (gene name abbreviations) were searched against a mapping file (a list of gene names, synonyms and abbreviations, *X*. *tropicalis* start and end position and length in bp) available from Xenbase [21], which allowed assignment to a specific gene and its genomic location in the *X*. *tropicalis*-genome. Different markers were then selected over the whole length of *X*. *tropicalis* linkage group 1. For comparative studies of sex chromosomes and autosomes, additional markers on several autosomal linkage groups were developed.

Because of the relatively low degree of polymorphisms in transcriptomic repeat sequences [7], different strategies were applied to increase the amount of polymorphic markers among candidates. Using Blast+ [18] with the megablast algorithm, candidate unigene sequences were searched against a database of the raw *B*. *viridis* Illumina reads. The sequences of the resulting Blast hits were mapped onto the unigene sequence using Geneious 7.06 (Biomatters). Ambiguities represented by a ratio of about 50%: 50% of raw Illumina reads in the resulting alignment were interpreted as heterozygous alleles in the individual diploid genome, from which the transcriptome was generated. It was expected, that markers, which were polymorphic within the single individual were likely to differ also between lineages. Furthermore, Serv [24] was applied, an algorithm, which uses a nonlinear model to calculate a numeric VARscore from intrinsic sequence properties. The VARscore predicts repeat variability and sequences, which were not already polymorphic within the single transcriptome, were selected for a high VARscore. Intron-exon boundaries of the repeat-containing unigenes were determined using the *X*. *tropicalis* genome [4] for these candidate genes using the Ensembl genome browser [25]. As amplification of genomic DNA markers fails if intron lengths and positions are not taken into account, *X*. *tropicalis* exons were aligned to the *B*. *viridis* unigene sequences using Geneious. PCR-primers for suitable marker candidates were then developed using Primer3 [26]. Primers were tested in 10 μl gradient PCR-reactions containing 1 μl *B*. *viridis* (or other green toads’) DNA (10 ng/μl), 1 μl 10x TopTaq-PCR-Buffer (with 15 mM MgCl_2_; Qiagen), 1 μl dNTP-Mix (2.5 mM of each dNTP), 0.5 μl forward primer (10 pmol/μl), 0.5 μl reverse primer (10 pmol/μl), 0.05 μl TopTaq polymerase (Qiagen) and 5.95 μl RNAse free water. The PCR protocol reads: initial denaturation at 95°C for 3 min, 35 cycles (including denaturation at 94°C for 30 s, annealing at 48–68°C for 60 s and elongation at 72°C for 40 s), and final elongation at 72°C for 10 min. PCR-products were screened for optimal annealing temperatures using agarose (2%) gel electrophoresis. Markers were tested for polymorphisms by direct sequencing of PCR-products and at least five individuals of four green toad species (*B*. *viridis*, *B*. *balearicus*, *B*. *siculus* and *B*. *variabilis*) were genotyped. For sequencing reactions, 25 μl PCR reactions were conducted, containing 2.5 μl DNA (10 ng/μl), 2.5 μl 10x TopTaq-PCR-Buffer (with 15 mM MgCl_2_; Qiagen), 2.5 μl dNTP-Mix (2.5 mM of each dNTP), 1.25 μl forward primer (10 pmol/μl), 1.25 μl reverse primer (10 pmol/μl), 0.125 μl TopTaq polymerase (Qiagen) and 14.875 μl RNAse free water. The PCR-protocol comprised initial denaturation at 95°C for 3 min, 35 cycles (including denaturation at 94°C for 30 s, annealing at optimal annealing temperature for 60 s and elongation at 72°C for 40 s), and a final elongation at 72°C for 10 min.

PCR-products were purified and Sanger sequenced by Eurofins MWG (Ebersberg, Germany) using one or both of the original PCR primers for sequencing reactions. Sequences were aligned to the transcriptomic unigene sequences using Geneious and manually checked for indels, i.e. desired length polymorphisms.

### Tests for sex linkage

To test whether markers located on *X*. *tropicalis* linkage group 1 are really located on the largely homologous *B*. *viridis* sex chromosome, sex-specific transmission was analyzed. In an XY-sex determination system, the father’s Y chromosome is always transmitted to male, while the X chromosome is always transmitted to female offspring. This was tested by genotyping previously generated genetic families with parents and offspring of known phenotypic sex [27, 28, 13]. Genotyping of all PCR-products was performed on an ABI 3500xL capillary sequencer (Applied Biosystems) with 0.5 μl diluted PCR-products in 9.25 μl Hi-Di formamide (Applied Biosystems) and 0.25 μl GeneScan 500 ROX size standard (Life Technologies). Genotypes were scored using Genemapper 4.1 (Applied Biosystems). Forward Primers of genotyping reactions were fluorescently labeled.

For markers BvCamk4 (ATTO-550 labeled), BvHNRNPD (FAM), BvCherp (ATTO-550), BvDMRT1 (HEX), BvVLDLR (HEX) and BvIno80b (HEX) genotypes of parents and offspring (28 females, 21 males) of *B*. *balearicus* x *B*. *siculus*-F_1_ Cr13 (father) and *B*. *balearicus* Si337 (mother) were genotyped. For markers BvEll2 (FAM) and BvGar1 (HEX) *B*. *balearicus* Si336 (father) and *B*. *siculus* Si335 (mother; [13]) and resulting offspring (n = 30) were genotyped. Because the phenotypic sex of this offspring was unknown, it was also genotyped with the classical microsatellite markers C223, known to be sex-linked in *B*. *siculus* and *B*. *balearicus* [28]. C223 was amplified using 4 μl RNAse free water, 3 μl Multiplex PCR kit master mix (Qiagen), 0.5 μl C223 forward primer (10 pmol/μl, FAM-labeled), 0,5 μl C223 reverse primer (10 pmol/μl) and 2 μl DNA (10 ng/μl). The PCR-protocol comprised: Initial denaturation at 95°C for 15 min, 35 cycles (denaturation at 94°C for 30 s, annealing at 55°C for 90 s and elongation at 72°C for 60 s), and final elongation was at 60°C for 30 min. 0.5 μl of the C223 PCR-products were diluted in 22 μl RNAse free water and genotyped. Marker BvChd1 is nested in the same intron for, which sex linkage has been shown [23].

### Cross amplification

Cross-amplification was tested for 11 markers located on *X*. *tropicalis* linkage group 1 (S3 Table). PCRs were performed with single high-molecular DNA samples of verified quality and quantity from nine systematic anuran families, namely *Allobates femoralis* (Arombatidae), *Bombina orientalis* (Bombinatoridae), *Bufo bufo*, *Rhinella marina* (Bufonidae), *Hyla arborea* (Hylidae), *Hyloxalus azureiventris* (Dendrobatidae), *Pelophylax esculentus*, *Rana temporaria* (Ranidae), *Pelobates fuscus* (Pelobatidae) and *Xenopus tropicalis* (Pipidae). Gradient-PCRs ranging in annealing temperatures from 47.8°C to 68°C were performed to determine the optimum. In case of uncertainties about quality, quantity or specificity of products, PCRs were repeated using a narrower gradient around the assumed optimal target temperature.

Cross-amplification was tested in 10 μl gradient PCR-reactions containing 2 μl DNA (10 ng/μl), 1 μl 10x TopTaq-PCR-Buffer (with 15 mM MgCl_2_, Qiagen), 1 μl dNTP-Mix (2.5 mM of each dNTP), 1 μl of 10x CoralLoad Concentrate (Qiagen), 1 μl of 5x Q-Solution (Qiagen), 0.4 μl of MgCl_2_ (25mM, Qiagen), 0.5 μl of each primer (10 pmol/μl), and 0.05 μl TopTaq polymerase (Qiagen) and 2.55 μl RNAse free water. We considered markers to be specific when gel electrophoresis showed a clear band in proximity to the fragment size of *B*. *viridis* used as positive control on the same gel with 1 kb DNA HyperLadder (Bioline). Double or multiple bands and much larger or smaller bands suggested unspecific amplification. If a band in the right size could be determined among multiple bands the marker was declared as inoperable. To determine exact marker sequences in *B*. *bufo*, we used the conditions described above for green toad sequences. Sequences of the markers which could be successfully cross-amplified in *B*. *bufo* were aligned to the *B*. *viridis* unigene using sequences homologous to *X*. *tropicalis*-LG1-genes. Mapping of the sequences was done in Geneious 7.0.6.

### Amplification success versus divergence time

To test inverse correlation of amplification success and evolutionary divergence, we took the divergence times of systematic anuran families from www.timetree.org. Amplification success is measured by the proportion of markers that amplified in each species to the total number of tested markers (S3 Table) developed for *B*. *viridis*. Maximum amplification success would be 10 out of 10 markers. Because of the proportional nature of the data, we calculated a generalized linear model (GLM) with binomial errors and a logit link (ANOVA, χ2). Statistical analysis was carried out in R version 3.1.0 [29].

## Results

### Transcriptome

The complete transcriptome data comprise 22,421,688 reads with a total length of 2,242,168,800 bp. The GC-content of the reads was 46.83%, 92.33% of all bases had a quality score of at least Q20. Assembly and scaffolding resulted in 110264 unigenes with a total length of 45,245,144 bp. Unigenes had an average length of 411 bp and a N50 of 504 bp. A total of 86707 unigenes ranged in length from 150 to 7508 bp (S1 Fig)

### Sequence Annotation

Sequence annotation revealed significant similarities to a large number of proteins from four protein databases (Table 1). Based on blast hits of the Nr database, 10541 unigenes (9.56%) were assigned to gene ontology terms.

**Table 1:** Unigenes with significant similarities to proteins from four different protein databases.

Database	Unigenes with significant similarity to Proteins	Blast hits with *E-values* lower than 1E-50
Swissprot	35692 (32.57%)	13130 (11.9%)
Nr	40193 (36.45%)	15220 (13.8%)
COG	9654 (8.75%)	1143 (1.03%)
Kegg	34322 (31.13%)	8418 (7.63%)

Regarding the molecular function ontology, the most common classifications were cellular processes (8156), metabolic processes (6713) and biological regulation. The most common categories for the cellular component ontology were cellular (10437), cell compartments (9976) and organelles (6529). In terms of the molecular function, most common categories involved binding (7609), catalytic activity (4663) and molecular transducer activity (897) (S2 Fig).

### Genes involved in sex determination or sex differentiation

Of a list 37 genes, ca. 87% (32) had significant blast hits in the *B*. *viridis* transcriptome, many of which providing the first sequence data for these genes in this non-model anuran species.

Compared to the length of *Xenopus* mRNAs used as BLAST templates, the majority of the *B*. *viridis* unigenes is shorter (between 10 and 92%) and likely represents fragments. In contrast, several unigenes are also longer, which may be indicative of insertions, for example by the extension of repetitive regions (Table 2).

**Table 2:** Male and female sex-determining and sex differentiation gene inventory and homologous *B*. *viridis* transcripts.

*Xenopus* protein sequence					*B*. *viridis* transcript			Relative length of *B*. *viridis* unigenes compared to *Xenopus* mRNA
Gene	Organism	Accession	Length of Protein (aa)	Length of mRNA (nt)	*B*. *viridis* unigene	*E-Value*	Length (nt)	
*AMH*	*X*. *tropicalis*	XP_004911480	609	2567	99159	1.97E-114	582	0.50
					10211	6.06E-026	690	
*AMHR2*	*X*. *tropicalis*	XP_012813307	527	1793	34820	6.63E-027	759	0.75
					99163	1.05E-11	582	
*AR*	*X*. *tropicalis*	XP_002941888	788	3497	91958	6.62E-145	583	0.17
*NR0B1*	*X*. *tropicalis*	XP_002933661	278	1298	-	-	-	-
*DHH*	*X*. *tropicalis*	NP_001090638	399	4372	77016	2.11E-7	303	0.07
*DMRT1*	*X*. *tropicalis*	XP_012808036	337	2444	106279	0	976	0.39
*DMRT3*	*X*. *tropicalis*	AEM44779	449	1416	55460	1.35E-48	227	0.16
*DMRT6 /DMRTB1*	*X*. *tropicalis*	XP_002931473	249	1849	106275	4.58E-31	975	0.53
*FGF9*	*X*. *tropicalis*	XP_002938621	208	928	89881	7.09E-119	408	0.44
*FGF16*	*X*. *tropicalis*	XP_012823079	202	609	34780	5.11E-51	185	0.30
*FGF20*	*X*. *tropicalis*	ACK58680	208	1490	23651	2.17E-11	160	0.11
*GATA-4*	*X*. *tropicalis*	NP_001016949	394	1599	8207	0	3517	2.20
*PDGFa*	*X*. *tropicalis*	NP_001163968	219	1574	81489	1.5E-75	330	0.34
					45059	4.3E-57	204	
*PDGFb*	*X*. *tropicalis*	AAI60575	240	2140	102381	4.44E-23	703	0.33
*SF-1*	*X*. *tropicalis*	NP_001139213	468	1407	109004	0	1537	2.70
					13031	0	2260	
*SOX8*	*X*. *tropicalis*	XP_002932315	466	2389	13157	9.79E-151	1118	0.56
					54344	3.67E-42	225	
*SOX9*	*X*. *tropicalis*	AAT72000	482	2538	109733	0	1967	0.78
*SOX10*	*X*. *tropicalis*	NP_001093691	436	2895	6192	6.63E-134	527	0.26
					56120	2.83E-56	229	
*SRD5A1*	*X*. *tropicalis*	NP_001006841	257	1537	99985	1.85E-72	607	0.64
					48658	6.04E-28	211	
					28798	2.24E-13	172	
*SRD5A2*	*X*. *tropicalis*	NP_001017113	239	1730	58235	2.57E-26	236	0.24
					34645	6.43E-14	185	
*SRD5A3*	*X*. *tropicalis*	AAH42255	319	1242	106318	1.8E-42	981	1.17
					10121	5.88E-17	469	
*WT1*	*X*. *topicalis*	NP_001135625	413	6193	-	-	-	-
*CYP26A1*	*X*. *tropicalis*	AAI71087	492	1458	15401	0	1252	0.86
*CYP26B1*	*X*. *tropicalis*	AAI35552	511	6137	13284	0	2396	0.39
*CYP26C1*	*X*.*tropicalis*	XP_002939137	533	4692	-	-	-	-
*CTNNB1*	*X*. *tropicalis*	NP_001016958	781	3382	104336	0	811	0.59
					107614	0	1171	
*ESR1A*	*X*. *laevis*	NP_001083086	585	-	-	-	-	-
*FOXL2*	*X*. *tropicalis*	XP_004917868	326	-	-	-	-	-
*RSPO-1*	*X*. *tropicalis*	NP_001121500	257	1946	82189	2.44E-29	1030	0.53
*WNT1*	*X*. *tropicalis*	XP_002935152	372	4323	103697	1.43E-121	770	0.25
					76924	1.18E-65	302	
*WNT4*	*X*. *tropicalis*	NP_001239015	351	1962	60581	5.21E-48	242	0.12
*ALDH1A2*	*X*. *tropicalis*	AAI57514	211	3696	57685	7.47E-63	234	0.06
*ALDH1A3*	*X*. *tropicalis*	XP_002939310	512	4056	99080	6.97E-133	579	0.26
					29168	1.44E-27	173	
					77287	4.42E-34	304	
*CXCR4B*	*X*. *laevis*	NP_001080681	358	2115	-	-	-	-
*HHIP*	*X*. *tropicalis*	NP_001007191	649	2717	14309	0	1001	0.65
					98123	5.23E-176	552	
					52556	1.96E-29	220	
*LRPPRC*	*X*. *tropicalis*	NP_001039203	1391	4347	15636	3.82E-82	2472	0.92
					108998	1.25E-170	1533	
*PTCH2*	*X*. *tropicalis*	XP_002937129	1423	6786	85924	6.32E-81	366	0.18
					53892	2.53E-54	223	
					38478	2.21E-42	193	
					92763	2.69E-41	447	

### Non-anonymous microsatellite markers and sex linkage

**Fig 1:**
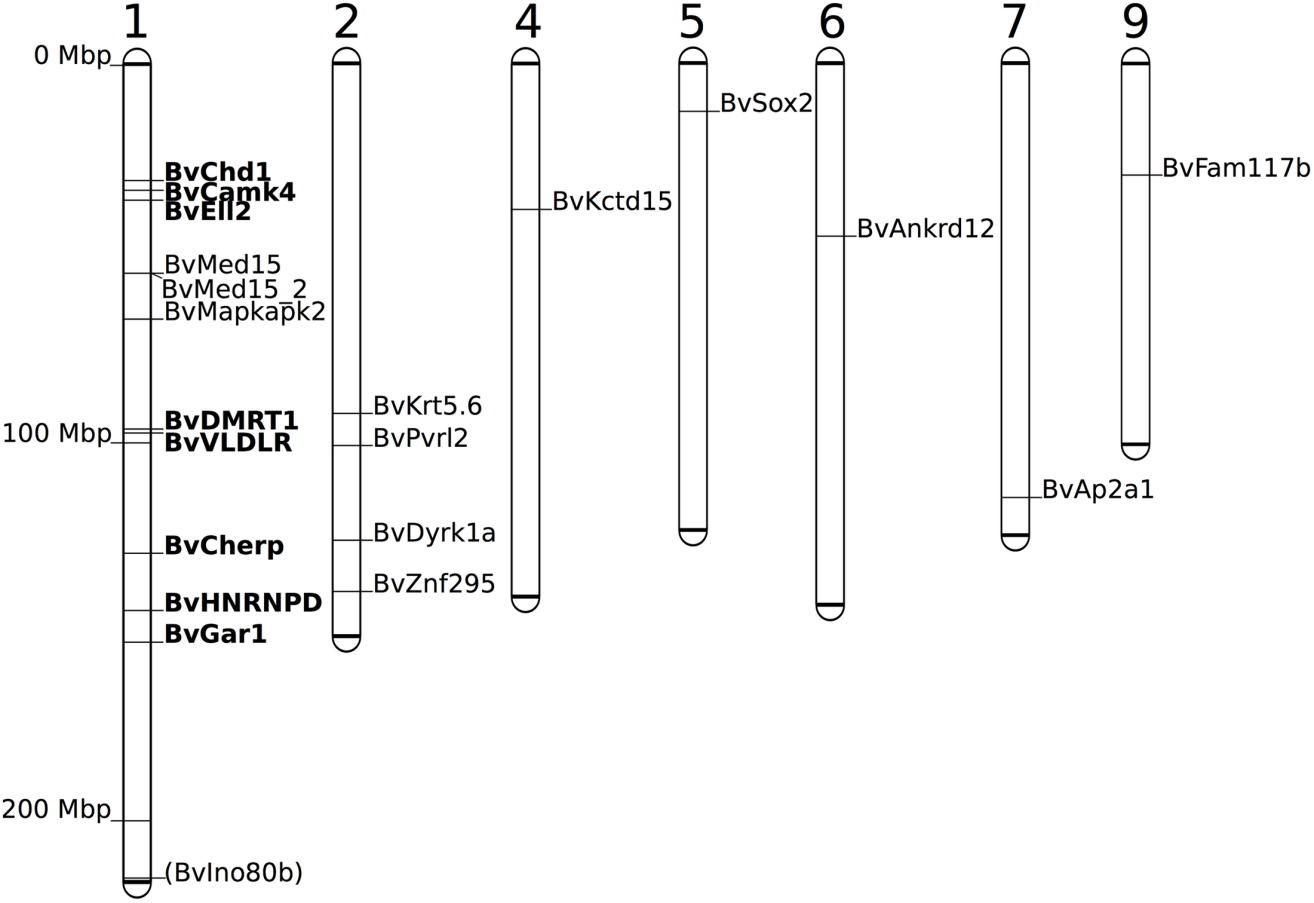
Genomic location of the newly developed transcriptome-based markers for Palearctic green toads (*B*. *viridis* subgroup) on *X*. *tropicalis* linkage groups. For markers written in **bold**, sex linkage was confirmed in green toads (main text for details). Marker names include the gene name abbreviation according to Xenbase. Linkage group size is relative to the proportion of the respective scaffold represented in the *X*. *tropicalis* genome v. 7.1 [4]. In green toads, “(Ino80b)” was found to be unlinked to other scaffold 1-homologous markers.

For comparative studies of sex chromosomes and autosomes, markers on several autosomal linkage groups, with emphasis on linkage group 2, were developed. In total, twenty-one microsatellite markers were generated from sequences with repetitive patterns found in the transcriptomic dataset (Fig 1, Table 3). Additionally, marker BvIno80b was generated to amplify an indel present in the transcriptome data. All of these microsatellites were polymorphic among *B*. *viridis*, *B*. *balearicus*, *B*. *siculus* and *B*. *variabilis*. Markers BvDMRT1, BvVLDLR and BvCHD1 were intron based indel-targeting markers, equally developed from transcriptome data by sequencing introns with primers matching flanking exons.

**Table 3:** Non-Anonymous microsatellite markers.

Name	Gene	*Xenopus*-LG	Position on *Xenopus*-LG	Fwd.-Sequence (5'-3')	Rev.-Sequence (5'-3')	Marker Type	*T*_*A*_ (°C)
BvCwc27	Cwc27	1	13842820–13932924	AAGTGATGACTTACGTAAAGAAGTACG	TCCTTTGCTTTTTCAATTTCTTTT	exonic-(TTCCTT)_n_-repeat	56
BvChd1	Chd1	1	30554621–30590231	TCCCTGCATTCTGTCAAGAAC	TGGACGGTATGAGGAACCAT	intronic-INDEL-targeting	66.1
BvCamk4	Camk4	1	33230874–33269817	CTCCTCTACCTCCCCGAGAT	CGGACTCCAGCTCGTAGTAA	exonic-(CTC)_n_-repeat	53.7
BvEll2	Ell2	1	36036707–36072713	GACTGAAACTGTCCATGGGC	TCTCATCTTACAAACAGGGTGC	exonic-INDEL-targeting	56.4
BvMed15	Med15	1	55139383–55188383	CAGCAAATGTCTCAACAAATGG	AGCAACTGCTGCTGACTCTG	exonic-(CAACAG)_n_-repeat	65
BvMed15_2	Med15	1	55139383–55188383	TTTCAGCAGCAGCAAGTAGC	GTTGCGGTTGCATCACATT	exonic-(CAG)_n_-repeat	65
BvPes1	Pes1	1	63613726–63635318	AGGAAGAAGACGAGGAGGATG	GCTCAGCAACTTGTGCTTTG	exonic-(GAG)_n_-repeat	68
BvMapkapk2	Mapkapk2	1	67440948–67481648	GTTCCAGTGAGCGGTGACT	CCAGGACTTTGCCGTTGATG	exonic-(CAG)_n_-repeat	56.4
BvDMRT1	DMRT1	1	96303907–96361376	AACTTCCCTGCAGACCTCCT	TGACTACTGGCAATTCATTCAT	intronic-INDEL-targeting	66.1
BvVLDLR	VLDLR	1	96940006–96981704	CCCTGTTAAGCATCAATTTGC	CTTGCAGTTCCTGCCAAGAT	intronic-INDEL-targeting	61
BvCherp	Cherp	1	129080135–129097850	GCCAATCCTTATGGCGTTCC	GCTGGTGGTTCGGGAATTG	Exonic-(AGCAACAGC)_n_-repeat	56.4
BvHNRNPD	HNRNPD	1	144610918–144625368	AGCGTGATGGAGGCGGCCGCCAGCG	CTTGCTGGCGTTGATCTTC	Exonic-(GCTTCT)_n_-repeat	59
BvGar1	Gar1	1	152691377–152698987	CTGGTGGTCCCTGGTCATAG	TTTAATCGGGGAGGAGGTGG	exonic-(TCC)_n_-Repeat	64.1
BvIno80b	Ino80b	1	215879679–215880220	ACTCCTGTGCTAAGACTCGC	GGATTGTGTAGGACGCCCC	INDEL-targeting in 3'-UTR	66.1
BvKrt5.6	Krt5.6	2	92165215–92171759	CAACACTGTATCCGCCTCCT	TGTCTGGAGAGATCACAAATCCA	exonic-(TCC)_n_ repeat	62.3
BvPvrl2	Pvrl2	2	100612435–100634031	ATAGCAGTCCCAGCAGAAGG	CTCATCCAAGGGCCAGAGA	(CT)_n_-repeat in 3'-UTR	66.2
BvDyrk1a	Dyrk1a	2	125823355–125869490	TGGGTTGGACTGTCACTGAA	GAATCTTGGGTGGAGGAGCT	exonic-(CAC)_n_-repeat	62.3
BvZnf295	Znf295	2	139330501–139336431	TGGAGTCACTTTCAGTCGCA	CTGGAACTGCTTTGCGTCTT	exonic-(CCA)_n_-repeat	64.5
BvKctd15	Kctd15	4	38249026–38281807	GGGCTTGTGAAACCAGAGG	ATCCCGGCCAGATCGTTT	exonic-(GAA)_n_-repeat	64.5
BvSox2	Sox2	5	12355967–12358300	TACATTCACGCAGCTTGGTG	GTCAAAGGCGCAGGATGAAA	(CT)_n_-repeat in 3'-UTR	66.2
BvAnkrd12	Ankrd12	6	45482374–45522238	AGAGCCACACTTCACCAACT	TGACCATATGCTTGTCTTCCA	exonic-(GAT)_n_-repeat	62.3
BvAp2a1	Ap2a1	7	114600274–114627891	GCAAACACTACAAGGAGGGTTT	TCTCTTGGACTTTACTGGACGT	(TG)_n_-repeat in 3'-UTR	57.5
BvFam117b	Fam117b	9	29130912–29142987	CAATTCACCCCGCAGCAG	CGGAGAGGTTGAGGTGGG	exonic-(CAC)_n_-repeat	63

Genes, their positions on *X*. *tropicalis* linkage groups, sequence of forward and reverse primers, marker type and optimal annealing temperature of the markers presented in this study.

Markers BvCamk4, BvDMRT1, BvCherp, BvHNRNPD and BvVLDLR show a signature of sex-specific transmission of alleles in the offspring of Si337 (mother, *B*. *siculus*) and Cr13 (father, *B*. *siculus* x *B*. *balearicus*). Sex-specific transmission is not perfect since single male specific alleles can be found in females and also single males without specific alleles are present. The marker BvIno80b showed no sex linkage in *B*. *viridis* and was therefore considered to be autosomal (S1 Table). For the sex-linked marker C223, allele 163 was perfectly linked to allele 225 of marker BvEll2, and allele 171 linked to allele 162 of the marker BvGar1 (S2 Table).

### Cross-amplification

**Fig 2:**
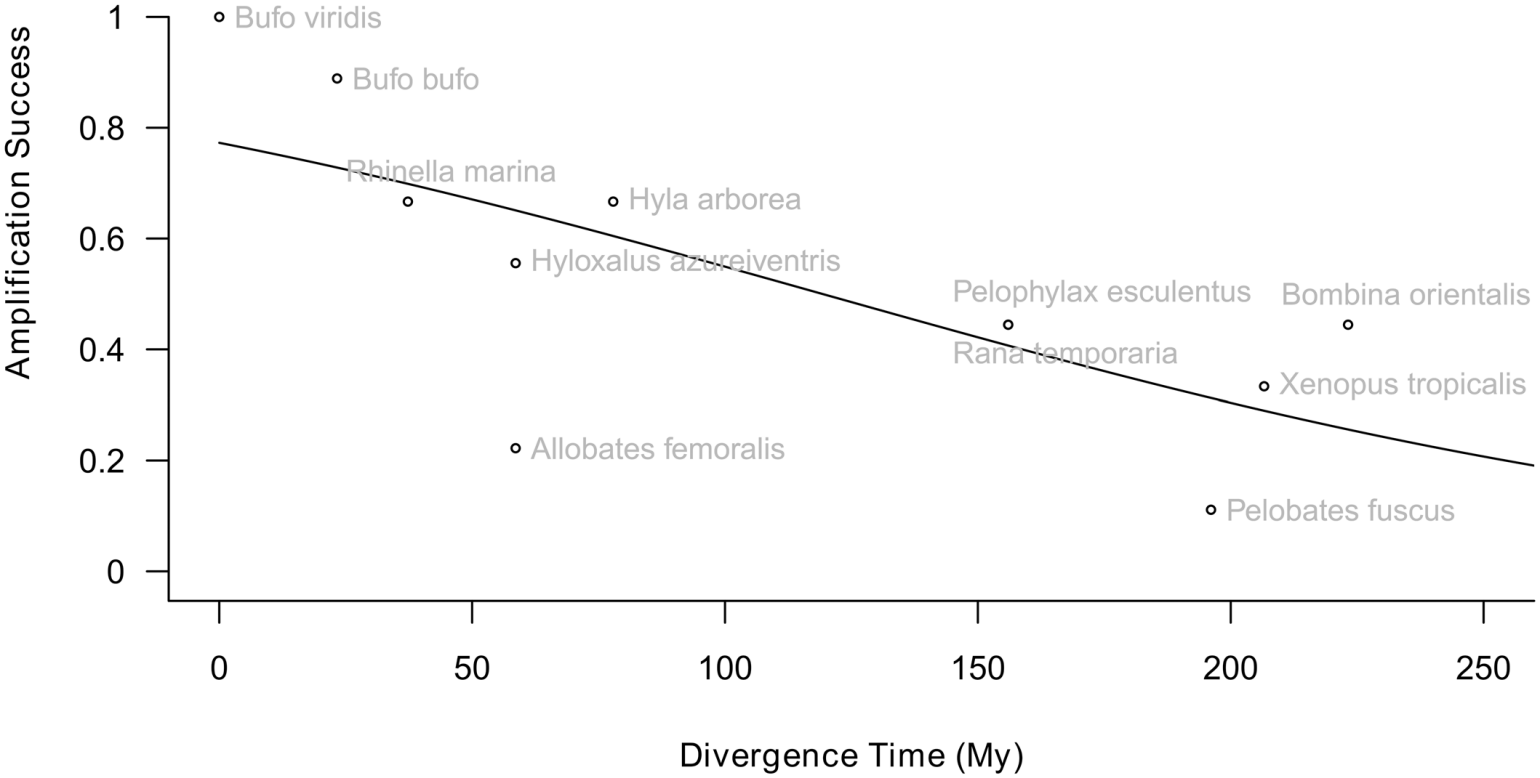
Amplification success plotted against divergence time in million years (My). The overall amplification success decreases with increasing evolutionary distance. Divergence time has a significantly negative effect on the cross-amplification success (df = 1, 9; deviance = 17.214, p < 0.001).

Cross-amplification trials in representatives from nine systematic anuran families showed variable success for different markers. The number of amplifying markers decreases with increasing evolutionary distance (Fig 2). In *B*. *bufo* (23.3 My divergence time to *B*. *viridis*) 9/11 markers amplified. In the second bufonid, *Rhinella marina* (37.3 My), 8/11 markers amplified. The amplification proportion for Dendrobatidae (58.6 My) was at a mean of 4.5/11 markers. For Hylidae and Ranidae (77.9 My) mean amplification success was 5.3/11 markers. In the most distant group consisting of Bombinatoridae and Pipidae (223.2 My) the mean amplification proportion was 3.5/11 and in *Pelobates fuscus* (Pelobatidae, 196.1 My) 2/11 markers amplified successfully.

## Discussion

The present study aimed at testing whether a single transcriptome of a non-model anuran species may present a reasonable resource for amphibians, where whole genome sequencing remains unaffordable due to large genome size. We have first tested this by retrieving a list of candidate genes, known for their role in sex determination and sex differentiation, and second by the targeted development of non-anonymous microsatellite markers. In terms of completeness, i.e. the presence of at least a fragment of a certain gene, our approach has provided reasonably sufficient data, as we could retrieve 87% of the desired genes. However, and somewhat expected, we face two problems regarding this list: i) deep divergences (ca. 200 My) to the model species may generally lower the blast hit success due to strong sequence divergence, ii) some expected sequences are either completely missing from the transcriptome or have become only available in small portions.

For the development of non-anonymous microsatellite markers, the single transcriptome has been useful. Since these markers are exonic (or UTR-linked), their genomic position can be mapped to the few available and only remotely related reference genomes. In this way, even in non-model species, genomic positions can be inferred based on structural conservation (synteny). Classical short polymorphic tandem-repeated DNA sequences (microsatellites) stem mostly from the non-coding, i.e. >98% of vertebrate genomes [30]. While these multiallelic markers can be easily genotyped, their genomic location usually remains unknown (“anonymous”). Thus, cross-amplification and consequently applicability to diverged lineages and species are often limited. Since exonic regions show a larger degree of sequence conservation, transcriptome-based microsatellites can ensure cross-amplification better than classical non-coding markers [31, 8]. Targeted development of green toad markers homologs on *X*. *tropicalis* LG 1, largely homologous to the *B*. *viridis* sex chromosome, has been successfully accomplished [23, 15]. For a subset of these markers, sex linkage could be demonstrated in genetic families: 7 out of 8 markers show expected sex-specific transmission in *B*. *viridis* (S1 and S2 Tables). Sex-specific transmission is not perfect in the genetic family Si337xCr13 (S1 Table), likely due to meiotic recombination along the *B*. *viridis* sex chromosomes, which has been shown in previous studies [28, 23]. Sex linkage of the 7 *B*. *viridis* microsatellites corroborates evidence for conserved synteny across the anuran LG 1 for Palearctic green toads. Only marker BvIno80b, located at the distal tip of *X*. *tropicalis* LG1 (Fig 1), shows no sex linkage, presumably a result of genome rearrangements.

The new transcriptome-based microsatellite markers also show a high cross-amplification success in several representatives across the anuran phylogeny. In other bufonids, *Bufo bufo* and *R*. *marina*, most markers could be successfully genotyped. Even in *Xenopus tropicalis*, a species with more than 200 My of divergence to *B*. *viridis*, three markers have still amplified successfully (S3 Table). Cross-amplification success inversely correlates with evolutionary distance [8]. Similar results have been shown for anonymous microsatellites in other vertebrate groups such as fish [32], crocodiles [33], birds [34, 35] as well as in other amphibians [36, 37]. Cross-amplification success among tree frogs declines by 50% after about 40 My [8]. The ‘half-life’ of our marker set tested for cross-amplification is about 120 My (predicted from regression). Despite the relatively small number of data points to test this relationship, our results suggest that cross amplification trials for transcriptome-based PCR products may be a convenient alternative for developing nuclear markers in anurans.

## Conclusions

Our data support the view that single, namely multiple-tissue based transcriptome sequencing is a reliable genomic resource and cost-efficient method for non-model amphibian species with large genome size and despite limitations, should be considered as long as genome sequencing remains unaffordable for most species.

## Supporting Information

S1 Fig Frequency length distribution of unigenes in the *B*. *viridis* transcriptome. (TIF)

S2 Fig Functional GO annotations of *B*. *viridis* transcriptome unigenes. (TIF)

S1 Table Sex linkage of markers BvCamk4, BvDMRT1, BvCherp, BvHNRNPD, BvIno80b and BvVLDLR. Genotypes of *B*. *balearicus* female (Si337) and F_1_-male (*B*. *balearicus x B*. *siculus*; Cr13) and their offspring. Phenotypic females are red colored, phenotypic males and alleles, which are expected to show sex-specific transmission, are marked in blue color.
(DOCX)

S2 Table Sex linkage of markers BvEll2 and BvGar1. Genotypes of Si335 and Si336 and the resulting offspring. Alleles, which show sex-specific transmission, are colored green and orange.
(DOCX)

S3 Table Markers developed for *B*. *viridis* that cross-amplified in the tested anuran species. In all ten examined species at least some of the markers showed bands in the agarose gel. Gradient PCRs were done with all markers to determine the optimal annealing temperature (*T*_*A*_) and the *T*_*A*_ range, in which amplification is possible. The approximate fragment length is estimated from the gel pictures.
(DOCX)
