# Supplementary figures and images for: A Single Transcriptome of a Green Toad (Bufo viridis) Yields Candidate Genes for Sex Determination and -Differentiation and Non-Anonymous Population Genetic Markers
Source: PLoS One. 2016 May 27;11(5):e0156419. doi: 10.1371/journal.pone.0156419 (PMC4883742; doi:10.1371/journal.pone.0156419)

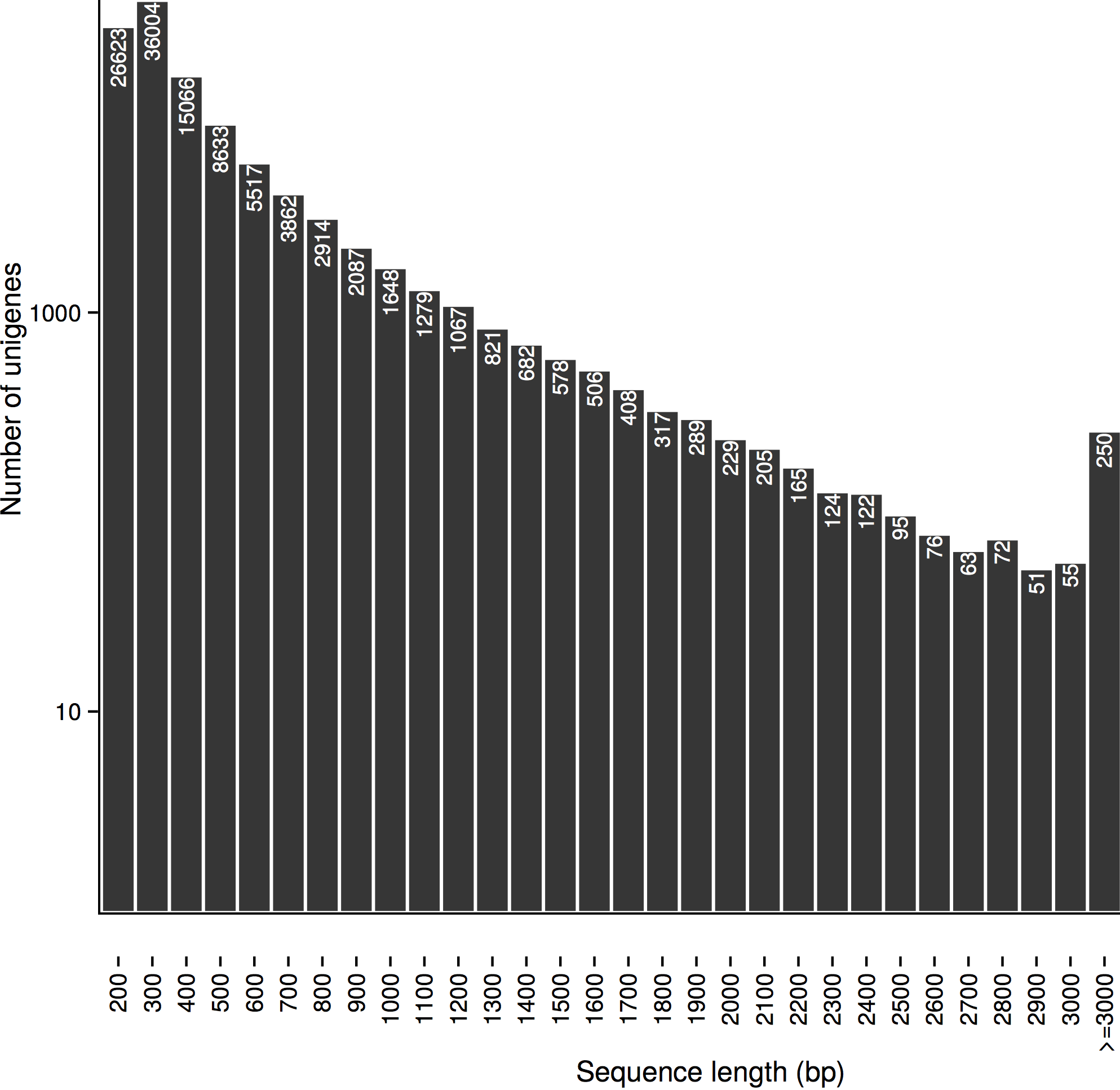

Supplement: S1 Fig — (TIF) [file pone.0156419.s001.tif]

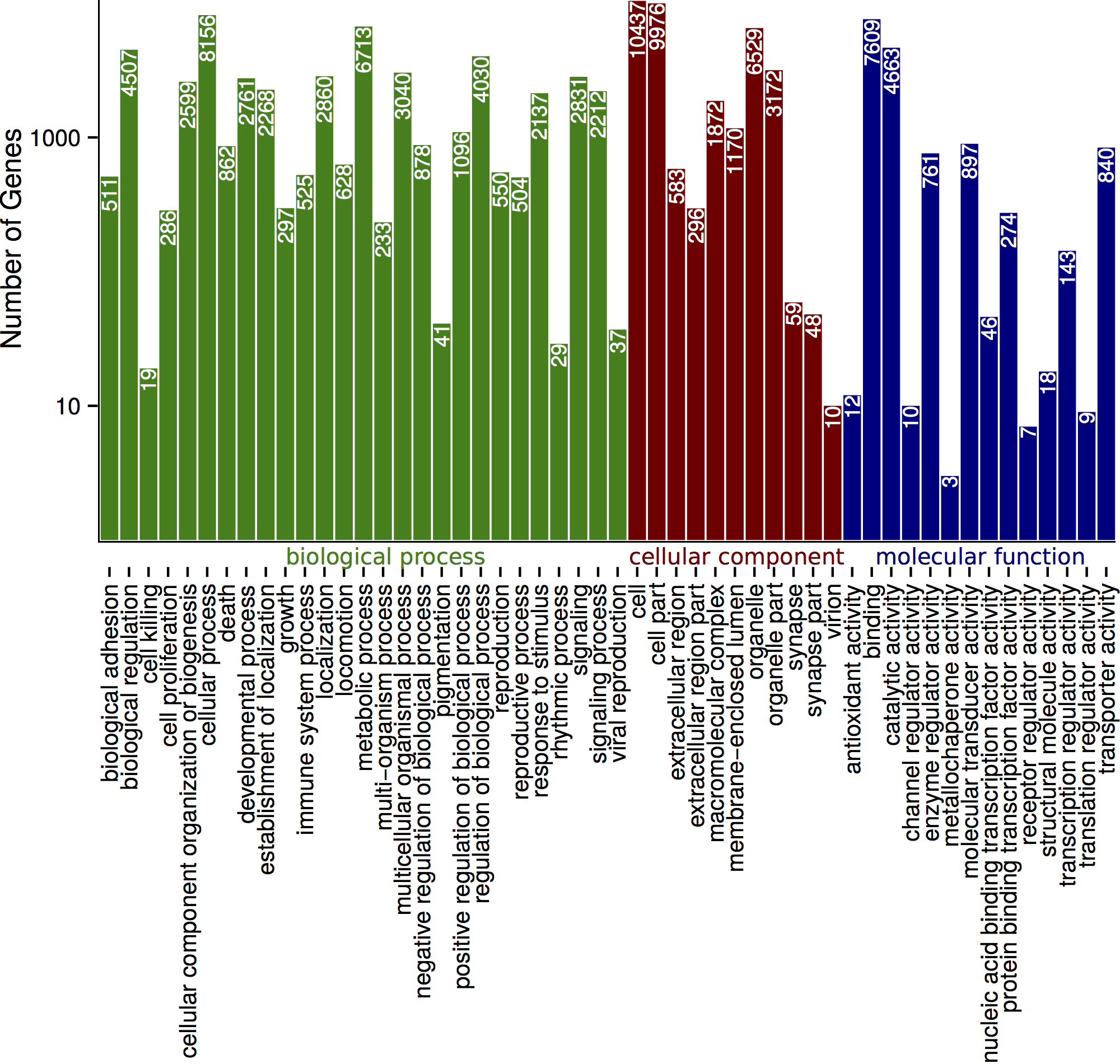

Supplement: S2 Fig — (TIF) [file pone.0156419.s002.tif]
