# Supplementary material for: A Single Transcriptome of a Green Toad (Bufo viridis) Yields Candidate Genes for Sex Determination and -Differentiation and Non-Anonymous Population Genetic Markers
Source: PLoS One. 2016 May 27;11(5):e0156419. doi: 10.1371/journal.pone.0156419 (PMC4883742; doi:10.1371/journal.pone.0156419)
Supplement: S1 Table — Genotypes of B. balearicus female (Si337) and F1-male (B. balearicus x B. siculus; Cr13) and their offspring. Phenotypic females are red colored, phenotypic males and alleles, which are expected to show sex-specific transmission, are marked in blue color. (DOCX) [file pone.0156419.s003.docx]

**S1 Table: Sex linkage of markers BvCamk4, BvDMRT1, BvCherp, BvHNRNPD, BvIno80b and BvVLDLR.**

| Individual | BvCamk4 | | | BvDMRT1 | | | BvCherp | | | BvHNRNPD | | | BvIno80b | | | BvVLDLR | |
| --- | --- | --- | --- | --- | --- | --- | --- | --- | --- | --- | --- | --- | --- | --- | --- | --- | --- |
| Si377 (mother) | 180 | 180 | 206 | | 206 | 166 | | 166 | 129 | | 129 | 210 | | 210 | 305 | | 305 |
| Cr13 (father) | 174 | 180 | 206 | | 229 | 163 | | 166 | 123 | | 129 | 202 | | 210 | 305 | | 316 |
| Si337xCr13 01 | 180 | 180 | 206 | | 206 | 166 | | 166 | 129 | | 129 | 202 | | 210 | 305 | | 305 |
| Si337xCr13 04 | 180 | 180 | 206 | | 206 | 166 | | 166 | 129 | | 129 | 202 | | 210 | 305 | | 305 |
| Si337xCr13 05 | 180 | 180 | 206 | | 206 | 166 | | 166 | 129 | | 129 | 210 | | 210 | 305 | | 305 |
| Si337xCr13 06 | 180 | 180 | 206 | | 206 | 166 | | 166 | 129 | | 129 | 202 | | 210 | 305 | | 305 |
| Si337xCr13 07 | 180 | 180 | 206 | | 206 | 166 | | 166 | 129 | | 129 | 202 | | 210 | 305 | | 305 |
| Si337xCr13 12 | 180 | 180 | 206 | | 229 | 163 | | 166 | 123 | | 129 | 210 | | 210 | 305 | | 316 |
| Si337xCr13 14 | 180 | 180 | 206 | | 206 | 166 | | 166 | 129 | | 129 | 210 | | 210 | 305 | | 305 |
| Si337xCr13 15 | 180 | 180 | 206 | | 206 | 166 | | 166 | 129 | | 129 | 210 | | 210 | 305 | | 305 |
| Si337xCr13 17 | 174 | 180 | 206 | | 206 | 166 | | 166 | 129 | | 129 | 210 | | 210 | 305 | | 305 |
| Si337xCr13 18 | 180 | 180 | 206 | | 206 | 166 | | 166 | 129 | | 129 | 202 | | 210 | 305 | | 305 |
| Si337xCr13 21 | 180 | 180 | 206 | | 206 | 166 | | 166 | 129 | | 129 | 202 | | 210 | 305 | | 305 |
| Si337xCr13 22 | 180 | 180 | 206 | | 206 | 166 | | 166 | 129 | | 129 | 202 | | 210 | 305 | | 305 |
| Si337xCr13 23 | 180 | 180 | 206 | | 206 | 166 | | 166 | 129 | | 129 | 210 | | 210 | 305 | | 305 |
| Si337xCr13 24 | 180 | 180 | 206 | | 206 | 166 | | 166 | 129 | | 129 | 202 | | 210 | 305 | | 305 |
| Si337xCr13 25 | 180 | 180 | 206 | | 206 | 166 | | 166 | 129 | | 129 | 202 | | 210 | 305 | | 305 |
| Si337xCr13 26 | 180 | 180 | 206 | | 206 | 166 | | 166 | 129 | | 129 | 210 | | 210 | 305 | | 305 |
| Si337xCr13 27 | 180 | 180 | 206 | | 206 | 166 | | 166 | 129 | | 129 | 202 | | 210 | 305 | | 305 |
| Si337xCr13 28 | 180 | 180 | 206 | | 206 | 166 | | 166 | 129 | | 129 | 210 | | 210 | 305 | | 305 |
| Si337xCr13 29 | 180 | 180 | 206 | | 206 | 166 | | 166 | 129 | | 129 | 210 | | 210 | 305 | | 305 |
| Si337xCr13 32 | 180 | 180 | 206 | | 206 | 166 | | 166 | 129 | | 129 | 202 | | 210 | 305 | | 305 |
| Si337xCr13 34 | 180 | 180 | 206 | | 206 | 166 | | 166 | 129 | | 129 | 202 | | 210 | 305 | | 305 |
| Si337xCr13 35 | 180 | 180 | 206 | | 206 | 166 | | 166 | 129 | | 129 | 210 | | 210 | 305 | | 305 |
| Si337xCr13 40 | 180 | 180 | 206 | | 206 | 166 | | 166 | 129 | | 129 | 210 | | 210 | 305 | | 305 |
| Si337xCr13 41 | 180 | 180 | 206 | | 206 | 166 | | 166 | 129 | | 129 | 202 | | 210 | 305 | | 305 |
| Si337xCr13 44 | 180 | 180 | 206 | | 206 | 166 | | 166 | 129 | | 129 | 210 | | 210 | 305 | | 305 |
| Si337xCr13 47 | 180 | 180 | 206 | | 206 | 166 | | 166 | 129 | | 129 | 202 | | 210 | 305 | | 305 |
| Si337xCr13 48 | 180 | 180 | 206 | | 229 | 163 | | 166 | 129 | | 129 | 210 | | 210 | 305 | | 316 |
| Si337xCr13 49 | 180 | 180 | 206 | | 206 | 166 | | 166 | 129 | | 129 | 202 | | 210 | 305 | | 305 |
| Si337xCr13 02 | 174 | 180 | 206 | | 206 | 166 | | 166 | 129 | | 129 | 202 | | 210 | 305 | | 305 |
| Si337xCr13 03 | 174 | 180 | 206 | | 206 | 166 | | 166 | 129 | | 129 | 202 | | 210 | 305 | | 305 |
| Si337xCr13 08 | 174 | 180 | 206 | | 229 | 163 | | 166 | 123 | | 129 | 210 | | 210 | 305 | | 316 |
| Si337xCr13 09 | 174 | 180 | 206 | | 229 | 163 | | 166 | 123 | | 129 | 202 | | 210 | 305 | | 316 |
| Si337xCr13 10 | 174 | 180 | 206 | | 229 | 163 | | 166 | 123 | | 129 | 202 | | 210 | 305 | | 316 |
| Si337xCr13 11 | 174 | 180 | 206 | | 229 | 163 | | 166 | 123 | | 129 | 202 | | 210 | 305 | | 316 |
| Si337xCr13 13 | 174 | 180 | 206 | | 229 | 163 | | 166 | 123 | | 129 | 202 | | 210 | 305 | | 316 |
| Si337xCr13 16 | 174 | 180 | 206 | | 229 | 163 | | 166 | 123 | | 129 | 210 | | 210 | 305 | | 316 |
| Si337xCr13 19 | 174 | 180 | 206 | | 229 | 163 | | 166 | 123 | | 129 | 210 | | 210 | 305 | | 316 |
| Si337xCr13 20 | 174 | 180 | 206 | | 229 | 163 | | 166 | 123 | | 129 | 210 | | 210 | 305 | | 305 |
| Si337xCr13 30 | 174 | 180 | 206 | | 229 | 163 | | 166 | 123 | | 129 | 202 | | 210 | 305 | | 316 |
| Si337xCr13 31 | 174 | 180 | 206 | | 229 | 163 | | 166 | 123 | | 129 | 202 | | 210 | 305 | | 316 |
| Si337xCr13 33 | 174 | 180 | 206 | | 229 | 163 | | 166 | 123 | | 129 | 202 | | 210 | 305 | | 316 |
| Si337xCr13 36 | 174 | 180 | 206 | | 229 | 163 | | 166 | 123 | | 129 | 202 | | 210 | 305 | | 316 |
| Si337xCr13 37 | 174 | 180 | 206 | | 229 | 163 | | 166 | 123 | | 129 | 202 | | 210 | 305 | | 316 |
| Si337xCr13 38 | 174 | 180 | 206 | | 229 | 163 | | 166 | 123 | | 129 | 202 | | 210 | 305 | | 316 |
| Si337xCr13 39 | 174 | 180 | 206 | | 229 | 163 | | 166 | 123 | | 129 | 202 | | 210 | 305 | | 316 |
| Si337xCr13 42 | 174 | 180 | 206 | | 229 | 163 | | 166 | 123 | | 129 | 202 | | 210 | 305 | | 316 |
| Si337xCr13 43 | 174 | 180 | 206 | | 229 | 163 | | 166 | 123 | | 129 | 202 | | 210 | 305 | | 316 |
| Si337xCr13 45 | 180 | 180 | 206 | | 229 | 163 | | 166 | 123 | | 129 | 210 | | 210 | 305 | | 316 |
| Si337xCr13 46 | 174 | 180 | 206 | | 229 | 163 | | 166 | 123 | | 129 | 202 | | 210 | 305 | | 316 |
