# Supplementary material for: A Single Transcriptome of a Green Toad (Bufo viridis) Yields Candidate Genes for Sex Determination and -Differentiation and Non-Anonymous Population Genetic Markers
Source: PLoS One. 2016 May 27;11(5):e0156419. doi: 10.1371/journal.pone.0156419 (PMC4883742; doi:10.1371/journal.pone.0156419)
Supplement: S2 Table — Genotypes of Si335 and Si336 and the resulting offspring. Alleles, which show sex-specific transmission, are colored green and orange. (DOCX) [file pone.0156419.s004.docx]

**S2 Table: Sex linkage of markers BvEll2 and BvGar1.**

|  | BvEll2 | | | BvGar1 | | | C223 | |
| --- | --- | --- | --- | --- | --- | --- | --- | --- |
| Si335 (mother) | 231 | 231 | 159 | | 159 | 179 | | 184 |
| Si336 (father) | 225 | 231 | 159 | | 162 | 163 | | 171 |
| Si335xSi336 1 | 231 | 231 | 159 | | 162 | 171 | | 179 |
| Si335xSi336 2 | 231 | 231 | 159 | | 162 | 171 | | 184 |
| Si335xSi336 3 | 231 | 231 | 159 | | 162 | 171 | | 184 |
| Si335xSi336 4 | 225 | 231 | 159 | | 159 | 163 | | 184 |
| Si335xSi336 5 | 231 | 231 | 159 | | 162 | 171 | | 179 |
| Si335xSi336 6 | 231 | 231 | 159 | | 162 | 171 | | 184 |
| Si335xSi336 7 | 231 | 231 | 159 | | 162 | 171 | | 179 |
| Si335xSi336 8 | 231 | 231 | 159 | | 162 | 171 | | 184 |
| Si335xSi336 9 | 225 | 231 | 159 | | 159 | 163 | | 184 |
| Si335xSi336 10 | 225 | 231 | 159 | | 159 | 163 | | 179 |
| Si335xSi336 11 | 231 | 231 | 159 | | 162 | 171 | | 171 |
| Si335xSi336 12 | 231 | 231 | 159 | | 162 | 171 | | 184 |
| Si335xSi336 13 | 231 | 231 | 159 | | 162 | 171 | | 179 |
| Si335xSi336 14 | 225 | 231 | 159 | | 159 | 163 | | 179 |
| Si335xSi336 15 | 231 | 231 | 159 | | 162 | 171 | | 184 |
| Si335xSi336 16 | 225 | 231 | 159 | | 159 | 163 | | 179 |
| Si335xSi336 17 | 225 | 231 | 159 | | 159 | 163 | | 179 |
| Si335xSi336 18 | 225 | 231 | 159 | | 159 | 163 | | 184 |
| Si335xSi336 19 | 225 | 231 | 159 | | 159 | 163 | | 179 |
| Si335xSi336 20 | 231 | 231 | 159 | | 162 | 171 | | 184 |
| Si335xSi336 21 | 231 | 231 | 159 | | 162 | 171 | | 179 |
| Si335xSi336 22 | 225 | 231 | 159 | | 159 | 163 | | 179 |
| Si335xSi336 23 | 231 | 231 | 159 | | 162 | 171 | | 184 |
| Si335xSi336 24 | 225 | 231 | 159 | | 159 | 163 | | 179 |
| Si335xSi336 25 | 225 | 231 | 159 | | 159 | 163 | | 179 |
| Si335xSi336 26 | 225 | 231 | 159 | | 159 | 163 | | 184 |
| Si335xSi336 27 | 225 | 231 | 159 | | 159 | 163 | | 179 |
| Si335xSi336 28 | 231 | 231 | 159 | | 162 | 171 | | 179 |
| Si335xSi336 29 | 225 | 231 | 159 | | 159 | 163 | | 179 |
| Si335xSi336 30 | 225 | 231 | 159 | | 159 | 163 | | 179 |
