# Supplementary material for: A Single Transcriptome of a Green Toad (Bufo viridis) Yields Candidate Genes for Sex Determination and -Differentiation and Non-Anonymous Population Genetic Markers
Source: PLoS One. 2016 May 27;11(5):e0156419. doi: 10.1371/journal.pone.0156419 (PMC4883742; doi:10.1371/journal.pone.0156419)
Supplement: S3 Table — In all ten examined species at least some of the markers showed bands in the agarose gel. Gradient PCRs were done with all markers to determine the optimal annealing temperature (TA) and the TA range, in which amplification is possible. The approximate fragment length is estimated from the gel pictures. (DOCX) [file pone.0156419.s005.docx]

**S3 Table: Markers developed for B. viridis that cross-amplified in the tested anuran species.**

| **Marker** | ***T_A_* range (°C)** | ***T_A_* optimum (°C)** | **Approx. Length. (bp)** |
| --- | --- | --- | --- |
| ***Allobates femoralis (Dendrobatidae)*** | | | |
| BvCherp | 48.3 – 51.4 | 51.4 | 150 |
| BvCwc27 | 56.4 | 56.4 | 80 |
| BvEll2 | 53.7 – 64.1 | 59.1 | 220 |
| ***Bombina orientalis (Bombinatoridae)*** | | | |
| BvMed15_2 | 49.5 – 53.7 | 53.7 | 250 |
| BvDMRT1 | 48.3 – 53.7 | 48.3 | 260 |
| BvVLDLR | 53.7 - 64.1 | 64.1 | 300 |
| BvMapkapk2 | 64.1 - 67.4 | 67.4 | 300 |
| ***Bufo bufo (Bufonidae)*** | | | |
| BvCherp | 47.8 – 67.4 | 64.1 | 300 |
| BvCwc27 | 47.8 – 56.4 | 51.4 | 70 |
| BvDMRT1 | 56.4 – 61.7 | 61.7 | 200 |
| BvEll2 | 47.8 – 67.4 | 67.4 | 190 |
| BvHNRNPD | 53.7 – 67.4 | 64.1 | 250 |
| BvIno80b | 53.7 - 67.4 | 67.4 | 200 |
| BvMapkapk2 | 47.8 – 64.1 | 64.1 | 300 |
| BvMed15 | 53.7 – 64.1 | 64.1 | 210 |
| BvPes1 | 51.4 – 67.4 | 59.1 | 300 |
| ***Rhinella marina (Bufonidae)*** | | | |
| BvCherp | 53.7 – 67.4 | 64.1 | 200 |
| BvCwc27 | 47.8 – 53.7 | 59.1 | 70 |
| BvDMRT1 | 56.4 – 61.7 | 53.7 | 190 |
| BvEll2 | 53.7 – 64.1 | 59.1 | 280 |
| BvIno80b | 53.7 | 53.7 | 280 |
| BvMapkapk2 | 47.8 – 64.1 | 64.1 | 270 |
| BvMed15 | 53.7 – 64.1 | 64.1 | 210 |
| BvPes1 | 47.8 – 51.4 | 49.5 | 250 |
| ***Hyla arborea (Hylidae)*** | | | |
| BvCherp | 48.3 – 49.5 | 49.5 | 170 |
| BvCwc27 | 53.7 | 53.7 | 100 |
| BvDMRT1 | 48.3 – 59.1 | 59.1 | 250 |
| BvEll2 | 53.1 – 64.1 | 64.1 | 240 |
| BvIno80b | 48.3 – 49.5 | 49.5 | 280 |
| BvMapkapk2 | 53.7 – 67.4 | 67.4 | 270 |
| BvVLDLR | 53.7 – 64.1 | 64.1 | 300 |
| ***Hyloxalus azureiventris (Dendrobatidae)*** | | | |
| BvChd1 | 47.8 – 49.5 | 47.8 | 290 |
| BvCwc27 | 53.7 – 59.1 | 59.1 | 80 |
| BvEll2 | 47.8 – 59.1 | 53.7 | 250 |
| BvMapkapk22 | 47.8 – 53.7 | 49.5 | 250 |
| BvMed | 49.5 – 59.1 | 59.1 | 200 |
| BvMed15_2 | 47.8 – 49.5 | 49.5 | 200 |
| ***Pelobates fuscus (Pelobatidae)*** | | | |
| BvChd1 | 48.3 | 48.3 | 300 |
| BvCwc27 | 53.7 – 59.1 | 59.1 | 80 |
| ***Pelophylax esculentus (Ranidae)*** | | | |
| BvCwc27 | 49.5 – 59.1 | 53.7 | 100 |
| BvEll2 | 47.8 – 53.7 | 53.7 | 230 |
| BvHNRNPD | 64.1 – 67.4 | 67.4 | 250 |
| BvMapkapk2 | 64.1 – 67.4 | 67.4 | 300 |
| BvVLDLR | 64.1 | 64.1 | 300 |
| ***Rana temporaria (Ranidae)*** | | | |
| BvCherp | 64.1 | 64.1 | 200 |
| BvEll2 | 59.1 – 64.1 | 64.1 | 250 |
| BvMapkapk2 | 64.1 – 67.4 | 67.4 | 300 |
| BvVLDLR | 59.1 – 64.1 | 59.1 | 300 |
| ***Xenopus tropicalis (Pipidae)*** | | | |
| BvHNRNPD | 49.5 – 67.4 | 67.4 | 250 |
| BvMapkapk2 | 64.1 – 67.4 | 67.4 | 280 |
| BvVLDLR | 53.7 – 64.1 | 67.4 | 300 |
